# History of pollutant adjuvants in respiratory allergy

**DOI:** 10.3389/falgy.2024.1374771

**Published:** 2024-03-12

**Authors:** Dennis Shusterman

**Affiliations:** Upper Airway Biology Laboratory, Division of Occupational, Environmental and Climate Medicine, Department of Medicine, University of California, San Francisco, CA, United States

**Keywords:** allergy, irritation, rhinitis, conjunctivitis, asthma, adjuvants

## Abstract

Combined exposures to allergens and air pollutants emerged as a topic of concern in scientific circles by the 1980's, when it became clear that parallel increases in respiratory allergies and traffic-related air pollution had been occurring during the 20th century. Although historically there has been a tendency to treat exposure-related symptoms as *either* allergic or toxicologic in nature, cross-interactions have since been established between the two modalities. For example, exposure to selected air pollutants in concert with a given allergen can increase the likelihood that an individual will become sensitized to that allergen, strongly suggesting that the pollutant acted as an ***adjuvant***. Although not a review of underlying mechanisms, the purpose of this mini-review is to highlight the potential significance of co-exposure to adjuvant chemicals in predicting allergic sensitization in the respiratory tract. The current discussion emphasizes the upper airway as a model for respiratory challenge studies, the results of which may be applicable—not only to allergic rhinitis—but also to conjunctivitis and asthma.

## 1 Introduction: allergy vs. irritation

*Allergy* denotes an acquired (and maladaptive) physical reaction to an otherwise innocuous foreign substance. It occurs in two phases—sensitization and challenge (i.e., “triggering”). An acutely triggered response to an allergen to which an individual is already sensitized is referred to as an *allergic reaction*, with the terms “allergy” and “allergen” normally being reserved for [Gell-Coombs] Type I (“immediate”) hypersensitivity. A hallmark of allergic sensitization is the presence of allergen-specific IgE in tissues, secretions, or in the circulation (1, 2). *Toxicologic irritation* (e.g., of the mucous membranes or airways), on the other hand, refers to the nociceptive and physiologic response to noxious chemicals, regardless of prior exposure history (3). While some primary sensations (symptoms such as itching) are more common with allergy than irritation, most secondary (“reflex”) symptoms—including nasal congestion, rhinorrhea, chest tightness, cough and wheezing—can occur with either phenomenon alone. When evaluating an individual case (or an aggregate population), the clinician (or epidemiologist) aiming to attribute symptoms to a specific mechanism may—however inadvertently—overlook *interactive effects* (4). In fact, the underlying mechanisms of allergy and toxicology can—and do—affect one another. Toxicologic potentiation of allergy is the topic of this brief review.

## 2 Air pollutants—definitions and categories

The term “air pollutant” refers to a range of potentially deleterious chemical substances found in outdoor, residential, or work atmospheres due to anthropogenic or natural emissions. Anthropogenic outdoor sources are conventionally classified as “stationary” (e.g., power plants or smelters), or “mobile” (motor vehicles), with vehicular emissions being a major concern. “Criteria Air Pollutants” (NO_x_, SO_x_, O_3_, CO, Pb, and particulate matter) are mandated for regulation as Ambient Air Quality Standards (AAQS) in the US (https://www.epa.gov/criteria-air-pollutants), leading to the establishment of ambient air monitoring networks in the US and elsewhere. Both ambient and indoor air may also include so-called “Toxic Air Contaminants” (or “TACs”), as defined in state and local standards (https://ww2.arb.ca.gov/resources/documents/carb-identified-toxic-air-contaminants). TACs include potential carcinogens, reproductive, or organ system toxicants. Indoor atmospheres can be influenced by both outdoor air and indoor sources [e.g., combustion appliances or (formaldehyde-containing) building materials]. Workplace air may contain any or all of the above substances, plus a wide range of industrial chemicals which may or may not have applicable workplace airborne exposure standards (see: https://www.osha.gov/annotated-pels and https://series.publisso.de/pgseries/overview/mak). Combustion processes (e.g., tobacco or cannabis smoking and various types of fires) can produce complex chemical mixtures. Despite this multiplicity of potential chemical exposures, data regarding interaction with allergens– which we summarize here—are available for only a small subset of chemicals.

## 3 Adjuvant effects of air pollutants

**Figure 1:**
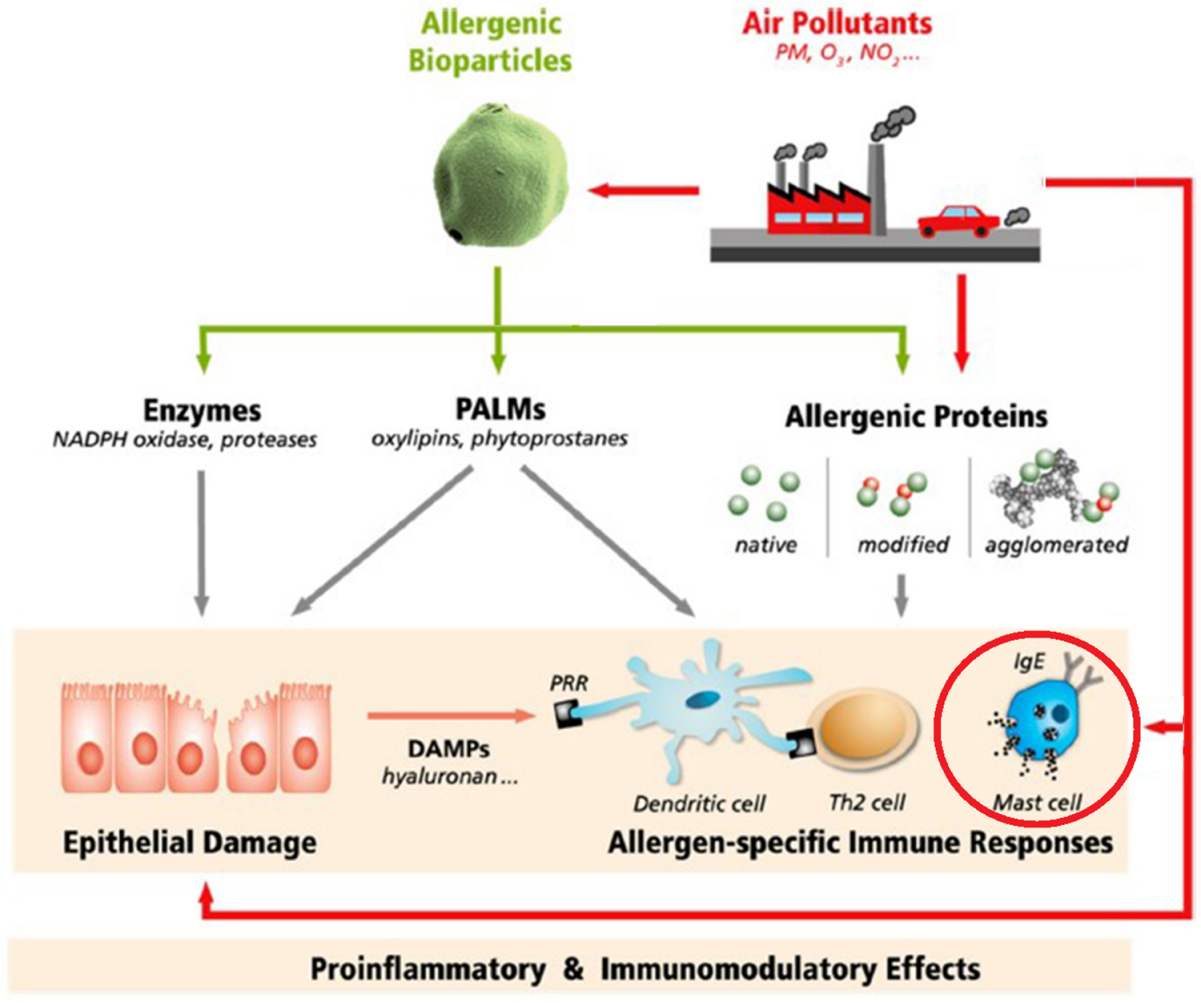
Potential mechanisms by which air pollutants may affect the response to airborne allergens. Air pollutants can interact physically and/or chemically with allergen particles, alter allergen penetration through the mucosa, interact with immune processes that elaborate allergen-specific immunoglobulin E (IgE), and/or carry out downstream effects of allergic triggering. PM, particulate matter; O_3_, NO*_x_*, ozone and nitrogen oxides; PALMs, pollen-associated lipid mediators; DAMPs, damage-associated molecular patterns; PRR, pattern recognition receptors; Th2, type 2 T helper cells (5).

In vaccine biology, the term “adjuvant” refers to a chemical added to a vaccine to increase its immunogenicity (hence, its effectiveness). Since the mid-1980's the phenomenon of adjuvancy has also been recognized in air pollution toxicology (in this case, chemical potentiation of allergen sensitization) (Figure 1). Air contaminants containing polycyclic aromatic hydrocarbons (PAHs, products of incomplete combustion of carbonaceous materials)—initially diesel exhaust particles (DEP)—were the first to be studied for their apparent adjuvant activity. In fact, the earliest published *in vivo* studies (in laboratory animals) came from Japan, where rapid industrialization and the proliferation of diesel-powered vehicles in the mid-20th century coincided with a significant uptick in observed respiratory allergies to aeroallergens—particularly to Japanese Cedar (6). Subsequent studies have built on this foundation.

### 3.1 Diesel exhaust particles

Beginning this area of experimental study, Muranaka and colleagues examined the effect of diesel exhaust particles (DEP) on immunologic sensitization to an allergen in female BDF_1_ mice. Using both ovalbumin (OVA) and Japanese Cedar Pollen as allergens of interest, aluminum hydroxide—Al (OH)_3_—as the positive control adjuvant, and intraperitoneal injection (IP) as the exposure system, the group showed that DEP was effective in boosting the specific IgE antibody response to allergen (7). The same group subsequently published a follow-up study showing an adjuvant effect of DEP on OVA sensitization when the two were co-presented to mice via *the intranasal route* (8).

Inspired by the above work, air pollutant adjuvants began to be examined *in vivo* using human volunteers by researchers in several countries. At the University of California (Los Angeles), Diaz-Sanchez and colleagues initially studied a mixed group of non-atopics and seasonal allergic rhinitics (studied outside of their relevant pollen season). They first found that direct application of DEP alone to the nasal mucosa elicited an increase in total IgE-secreting cells in nasal lavage (NL) fluid (9). Studying a similar volunteer group, the investigators later found that nasal DEP administration alone enhanced pro-inflammatory cytokine expression in NL fluid (10). The same group subsequently documented both DEP-induced augmentation of ragweed-specific IgE after allergen challenge of sensitized individuals (11) and IgE isotype-switching *in vivo* (12).

Against this backdrop, a major breakthrough was achieved in 1999 by researchers pairing DEP exposure with exposure to an antigenic substance to which casual contact is unlikely in the general population—keyhole limpet hemocyanin or “KLH”—a substance found in the endolymph of a marine mollusk (13). Using KLH as the allergen, researchers studied seasonal allergic rhinitics by applying the substance topically in the nose with and without DEP pre-treatment (14). Among the 10 subjects exposed to the allergen alone, none had detectable anti-KLH IgE in NL fluid post-exposure. Among the subjects treated with both KLH and DEP, on the other hand, 9 of 15 showed detectable levels of KLH-specific IgE (14). Following this dramatic finding, respiratory adjuvant studies gained further momentum. Investigators in Japan, for example, using cultured human nasal epithelial cells, documented that DEP alone (unpaired with allergen) upregulates messenger RNA for histamine receptors, as well as increasing histamine-induced IL-8 production (15). Back at the University of California, the Diaz-Sanchez group studied basophils from allergic rhinitic and control subjects *in vitro*, showing that DEPs alone (without allergen) could release IL-4 and histamine from these cells regardless of the allergy status of their donors (16).

At this point another research thread developed, regarding the constituents of DEP (itself a complex mixture of ingredients). In 2000, the Diaz-Sanchez group pre-treated dust mite (*Der p 1*)—sensitive allergic rhinitics with either DEP or carbon black (pure carbon particles) and found that DEP—but not carbon black—augmented their symptoms, lowered their threshold for response, and increased IL-4 and histamine after allergen triggering (17). In 2002, researchers at the Pasteur Institute in Paris compared the adjuvant effect of traffic particulate matter (TPM) with that of pure carbon core particles, and found that only TPM boosted levels of birch pollen-specific IgE and eosinophils (18). The following year researchers in the Netherlands published a wide-ranging study in which both mice and rats were sensitized (to timothy grass and OVA, respectively), and five different particulate matter sources were used. The mouse / OVA system had a lower threshold for response and was able the rank the different PM sources by potency (19). In 2007 group in Germany incubated basophils from birch pollen-allergic donors in the presence of two different PAH compounds—benzo[a]pyrene (BaP) and phenanthrene (Phe)—then stimulated the cells with birch allergen (*rBet v 1*). They found that combining either of these DEP constituents with allergen significantly increased basophil activation over that of allergen alone (20).

### 3.2 Second-hand [tobacco] smoke

Coincident with the above work, a parallel line of inquiry emerged concerning a different product of incomplete combustion—so-called “second-hand smoke” (“SHS”). Like DEP, SHS contains PAHs (i.e., known adjuvants). A link between childhood SHS exposure and persistent wheeze / asthma had been documented by the late 1980's, but differentiating between irritant and adjuvant effects remained ambiguous (21–23). It was only once biomarkers of *both* SHS exposure (cotinine) *and* allergic sensitization (specific IgE) were asayed from the same individuals that the likely role of SHS as an adjuvant was established (24, 25).

In 1997, Seymour and colleagues, from the University of California (Davis), sensitized mice to OVA by intraperitoneal injection (with and without pre-exposure to SHS), with subsequent OVA aerosol exposure. The mice who were pre-exposed to SHS displayed an augmented biochemical response to allergen provocation, including higher *total* IgE and OVA-specific IgG1 (26). In 2001, SHS was employed experimentally by the UCLA group to exhibit an OVA-*specific* IgE adjuvant effect via airborne exposure—along with OVA—in two strains of mice (27), a finding replicated in Belgium some 5 years later (28). 2006 would also see the publication of the first human study involving combined SHS + allergen exposures. Diaz-Sanchez and colleagues nasally challenged 19 nonsmoking, ragweed-sensitized allergic rhinitics with either ragweed pollen extract (containing *Amb a* 1) or placebo. These challenges occurred immediately after the subjects had spent 2 h in a chamber with either clean air (mean particulate concentration 46 μg/m^3^) or SHS (mean particulate concentration, 310 *μ*g/m^3^). Significantly, the combined SHS-allergen exposure yielded a ∼16-fold greater increase in ragweed-specific IgE in NL fluid (compared to the clean air-allergen exposure) when subjects were sampled 4 days later. In addition, IL-4, IL-5 and IL-13 (all Th2-cytokines) and histamine were increased after SHS-allergen exposure, and IFN-γ (a Th1 cytokine) was suppressed (29).

In 2008 Samuelsen et al. in Norway, combining subdermal injection of OVA in Balb/cA mice with selected particles, compared the IgE-related adjuvant activity of particles from woodsmoke and road traffic with that of DEP. They found that woodsmoke and road traffic particles were roughly equipotent, although still less effective than DEP (30). A 2009 mouse study conducted at UCLA revealed that ultrafine PM (<0.15 μm) was a more potent adjuvant than was fine PM (<2.5 μm) (31). More recently, Castaneda and colleagues (2018) studied the adjuvant properties of atmospheric fine particulate matter (<2.5 μm) collected near a major highway in an asthma-prevalent area of California, finding that pairing house dust mite (HDM) inoculation with PM in a murine model substantially enhanced both pulmonary IgE elaboration and inflammation relative to HDM exposure alone (32). Most recently in Shanghai, a 2023 study characterized pulmonary lymph nodes in mice sensitized to HDM found that co-exposure to PM2.5 increased both IgE production and associated cellular inflammation (33).

### 3.3 Inter-individual variability in the adjuvant response

As noted above in the discussion of DEP adjuvancy, *de novo* sensitization of atopic human subjects to KLH in the presence of DEP was not a universal outcome, given that 6 of 15 subjects receiving both allergen and adjuvant failed to show sensitization. The UCLA group revisited this anomaly initially in 2003 by comparing 18 ragweed-allergic volunteer subjects' responses to either DEP + *Amb a 1* or Placebo + *Amb a 1* in a randomized, counter-balanced, replicate provocation study. Examining [allergen-specific] IgE, IL-4 and IFN-γ in NL fluid, they found that the net response to allergen challenge was both highly variable between—and highly reproducible within—subjects. They concluded that susceptibility to the adjuvant effect of DEP was and “intrinsic trait” rather than a random phenomenon (34).

The following year the same group followed-up with another comparison of DEP vs. Placebo pre-treated [ragweed-sensitized] subjects, adding both histamine as an analyte and an assay for possible variants in their glutathione-S-transferase enzymes (i.e, GSTM1, GSTP1, and GSTT1 genotypes). They found that subjects that were GSTM1 null, as well as those homozygous for the GSTP1 I105 (wildtype) genotype, displayed a more robust IgE response (and greater histamine production) than their genetic counterparts. The authors concluded that the inter-individual differences previously documented in DEP adjuvant studies were—in large part—mediated by differences in enzymatic reduction of “reactive oxygen species,” with the more effective glutathione-S-transferase genotypes conferring a protective effect against DEP (35). Similar results were obtained when SHS was substituted for DEP in an equivalent study (36).

Successful interruption of DEP-mediated adjuvancy via administration of exogenous antioxidants has also lent credibility to the “oxidative stress” model of adjuvant action. Whitekus et al. screened six candidate antioxidants—two botanicals (silibinin and luteolin), two vitamins (C and E), and two sulfur-donors (Bucillamine [or BUC] and N-acetylcystine [or NAC])—for their ability to interrupt DEP-induced adjuvancy when co-administered with OVA (37). The last two (both sulfur-donors) were highly efficacious in that role. As an alternative to exogenous supplementation, induction of phase II metabolic enzymes has since been shown to exert an equivalent antioxidant role. For example, in 2007, Ritz et al. added sulforaphane (a natural compound derived from cruciferous vegetables) to cultured bronchial epithelial cells and observed inhibition of DEP-stimulated production of the proinflammatory cytokines IL-8, GMCSF, and IL-1β (38). *In vivo*, this effect was later replicated in a nutrition study in which broccoli sprout extract was administered to human subjects in mango juice on a daily basis (39).

### 3.4 Adjuvant assays of other chemicals

In addition to PAH-containing combustion products (DEP, SHS and woodsmoke), other commonly encountered chemical compounds have been assayed with respect to their potential role as adjuvants. These include formaldehyde (a common indoor air pollutant), phthalates (found in plastics), the industrial chemicals styrene, chloroform, and 1,1-dichloroethylene, and the reaction products of ozone and limonene (an unsaturated terpene used in some cleaning products).

#### 3.4.1 Formaldehyde

Studies of the potential effect of formaldehyde (CH_2_O) vapor on allergic sensitization date back at least to the mid-1990s. Riedel and colleagues pre-exposed Dunkin-Hartley guinea pigs to clean air, 0.13 ppm, or 0.25 ppm CH_2_O vapor on a daily basis for 5 days, followed by aerosolized OVA after pre-exposure and 2 weeks later. After another week's time the animals underwent venipuncture and bronchoprovocation with OVA. In the 0.25 ppm pre-exposed group 10/12 animals (vs. 3/12 in the control group) exhibited a positive provocation reaction to OVA. The median OVA-specific IgG1 level was also elevated in the 0.25 ppm group compared to the controls (specific IgE was, however, not assayed). The 0.13 ppm exposure group showed intermediate reactivity to OA on pulmonary function and also an intermediate distribution of IgG1 antibody titers (40).

Two additional studies on formaldehyde were published during the period 2009–2011 using CH_2_O exposures comparing two different Chinese workplace exposure standards (which had recently been reduced∼6-fold from 2,500 to 400 ppb). Qiao and colleagues found that CH_2_O at the higher concentration increased bronchial reactivity in rats, both alone (a possible irritant effect) and even more so with OVA sensitization (i.e., as an adjavant) (41). Similarly, Liu et al. found that co-exposure of mice to CH_2_O at the higher standard in concert with OVA sensitization greatly amplified bronchial reactivity vs. clean air or CH_2_O at the lower standard (42).

#### 3.4.2 Phthalates

Similar to DEP, potential exposures to phthalate plasticizers increased dramatically in the 2nd half of the 20th century, with increased use of plastics in consumer products coinciding with the observed increase in the prevalence of airway allergies. As a result, this class of chemicals was also suspected to be an adjuvant for allergic sensitization. Since most phthalates have very limited volatility, the majority of the early phthalate assays involved either parenteral administration or gavage. Of these studies, some *did* indicate that phthalates could facilitate allergic sensitization (including elaboration of allergen-specific IgE) (43, 44). In a later set of studies, phthalates were co-administered with a test allergen (OVA) via the *inhalation* route. Two such studies looked at di-ethylhexyl phthalate (DEHP) and its chief metabolite, mon-ethylhexyl phthalate (MEHP), utilizing OVA as the allergen. *Both* phthalates boosted allergen-specific IgG1, but *neither* significantly affected allergen-specific IgE levels (45, 46).

#### 3.4.3 Industrial chemicals

Ban and colleagues studied the effect of co-exposure to three commonly used industrial chemicals:—1,1-dichloroethylene (DCE), chloroform (CF), and styrene (S)—during OVA sensitization in mice. The endpoints studied were all biochemical in nature, including OVA-specific IgE in blood, and cytokine production in lung-draining lymph nodes. The authors found that of the three, only chloroform and styrene co-exposures reliably potentiated the generation of allergen-specific IgE. Further, of these two, only styrene produced an expected boost in Th2 cytokine production (47).

#### 3.4.4 Reaction products of ozone and limonene

**Figure 2:**
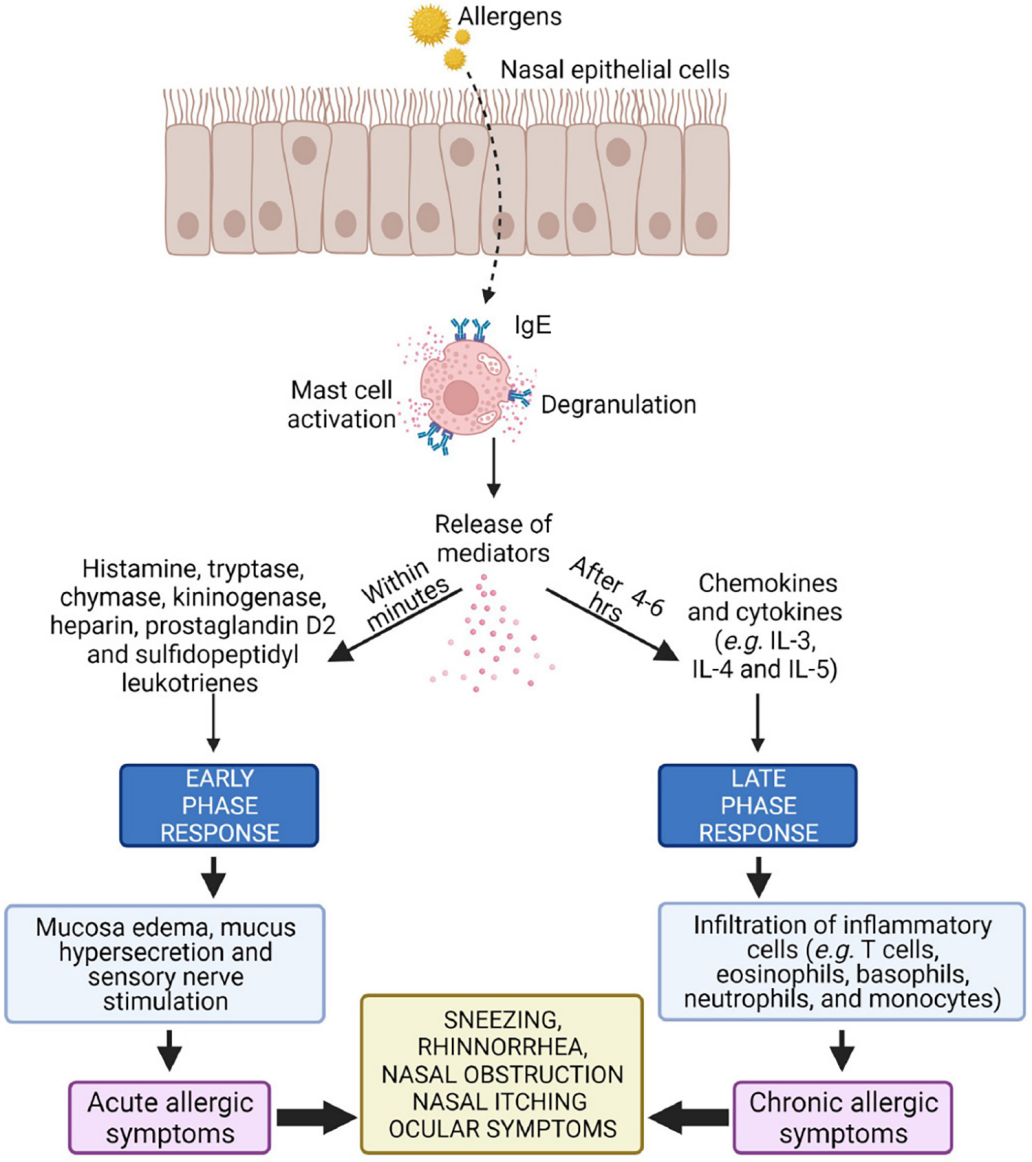
Activation (“triggering”) of a sensitized mast cell in the nasal mucosa, a prerequisite for which is the presence of allergen-specific IgE. Two reaction kinetics are delineated, “early” (minutes) and “late” (>4–6 h), with distinct biological markers associated with each. Differences in cellular / biochemical outcomes between otherwise similar studies may result from sampling time differences (49).

Hansen et al. compared OVA-specific Ab's in BALB/cJ mice exposed to OVA alone (negative control), OVA with Al(OH)_3_ (positive control), OVA with O_3_, OVA with limonene (a cyclic terpene), and OVA with reaction products of O_3_ + limonene. Besides the positive control, only the combination of OVA with reaction products of O_3_ + limonene boosted allergen-specific IgE compared with the negative control. *Of note, the authors stopped short of labeling this an “adjuvant effect,” since airway inflammation was not evident when bronchoalveolar lavage fluid was obtained from the animals' lungs ex vivo* (48) Figure 2.

## 4 Discussion

Over the last three decades, considerable attention has been devoted to studying the effects of air pollutants on respiratory allergies (particularly adjuvant effects). Candidate adjuvants have mostly been identified based on epidemiologic correlations. Specifically, in the 20th century an increasing prevalence of respiratory allergies was observed coincident with increases in: (1) traffic-related air pollutants (e.g., DEP), (2) exposure to “second-hand” tobacco smoke, (3) use of formaldehyde-releasing resins in building materials, and (4) use of phthalates in consumer products. For the first three examples (DEP, SHS and CH_2_O), combined inhalation exposures confirmed adjuvancy. For the fourth, effective adjuvant doses would require ingestion of the phthalate in concert with allergen inhalation.

Approaches to studying adjuvant effects have included *in vitro* (cell culture) and *in vivo* (animal and human) studies. The most impressive of these featured atopic human volunteers who were successfully sensitized to what at the time was termed a “neoallergen” (KLH). At the opposite end of the spectrum, a number of ostensible “adjuvant” studies have dealt with biochemical endpoints *without* allergen-specific IgE determination. These diverse results can be challenging to fit into a single mechanistic model, and after more than three decades of study, we are still struggling to put the “puzzle pieces” together.

### 4.1 Future directions in adjuvant research

In terms of completed studies, some methodologic issues are readily apparent (albeit not conspicuously highlighted in the published literature). Foremost among these is the fact that a subset of authors hold that—in addition to a candidate adjuvant chemical needing to facilitate formation of allergen-specific IgE—it would also need to produce elevated biochemical markers of tissue inflammation post-triggering. Since this criterion is inconsistently applied, establishing guidelines regarding this requirement would hopefully reduce the level of ambiguity in interpreting future study findings.

Another potential contemporaneous theme in adjuvant research would take its inspiration from early work in which the effects of DEP (vs. pure black carbon particles) were compared, finding that adjuvancy (increased elaboration of anti-OVA IgE + IgG) was confined to the former (17). Surprisingly, Granum and colleagues subsequently demonstrated an adjuvant effect when pairing allergen exposures with other pure “model” particles (composed of a variety of materials including polystyrene, amorphous silica, titanium dioxide, and tetrafluoroethylene or Teflon). Granum also highlighted the fact that “ROFA” (residual oil fly ash), which is produced under similar conditions as DEP, only exhibits adjuvancy when selected metals (i.e., nickel or vanadium—but not iron) are adsorbed thereto (50). Implicit in these findings is the fact that—mechanistically—the physical chemistry of a candidate adjuvant must be taken into account and reconciled with the prevailing model emphasizing oxidative stress as a common factor.

Numerous review articles deal with pollutant adjuvants, although in most cases in a wider context of chemically induced inflammation (5, 49, 51–55). Research into pollutant adjuvant effects would benefit from a more stringent definition of the phenomenon, as well as clearer boundaries with respect to related—but distinct—studies of xenobiotic-induced inflammation. Once the term “adjuvant” stands out as a distinct entity with well-defined borders, further progress will likely follow.
